# Supplementary material for: Accurate Three-Dimensional Thermal Dosimetry and Assessment of Physiologic Response Are Essential for Optimizing Thermoradiotherapy
Source: Cancers (Basel). 2022 Mar 27;14(7):1701. doi: 10.3390/cancers14071701 (PMC8997141; doi:10.3390/cancers14071701)
Supplement: Supplementary file 1 [file cancers-14-01701-s001.zip › cancers-1601092-supplementary.pdf]

cancers

MDPI

Supplementary Material: Accurate Three-Dimensional Thermal Dosimetry and Assessment of Physiologic Response Are Essential for Optimizing Thermoradiotherapy

Mark W. Dewhirst, James R. Oleson, John Kirkpatrick and Timothy W. W. Secomb

Table S1. Temperatures obtained during 1st HT: Thermal dose fractionation trial ^.

Variable	Treatment Group	Median
T10	5 Fx HT	45.1 °C
	20 Fx HT	43.4 °C
T50	5 Fx HT	43.1 °C
	20 Fx HT	41.3 °C
T90	5 Fx HT	41.0 °C
	20 Fx HT	40.0 °C
Duration of 1st HT (min)	5 Fx HT	45 min
	20 Fx HT	90 min

N = 21 for 5 Fx HT group; *N* = 16 for 20 Fx HT group; ^ Data from all subjects entered into the trial.

Table S2. Key Thermal Characteristics: Thermal dose fractionation trial ^.

Variable	Treatment Group	Median (min)	Thermal Dose Rate #
Total $CEM43T_{10}$	5 Fx HT	750	3.64
	20 Fx HT	1814	1.20
Total $CEM43T_{50}$	5 Fx HT	201	1.00
	20 Fx HT	291	0.19
Total $CEM43T_{90}$	5 Fx HT	29.9	0.15
	20 Fx HT	24.9	0.016
Duration of HT (min)	5 Fx HT	206	N/A
	20 Fx HT	1506	N/A

N = 21 for 5 Fx HT group; *N* = 16 for 20 Fx HT group; ^ Data from all subjects entered into the trial; # $CEM43T_x$/min; Thermal dose rate = total $CEM43T_x$/total duration of HT.

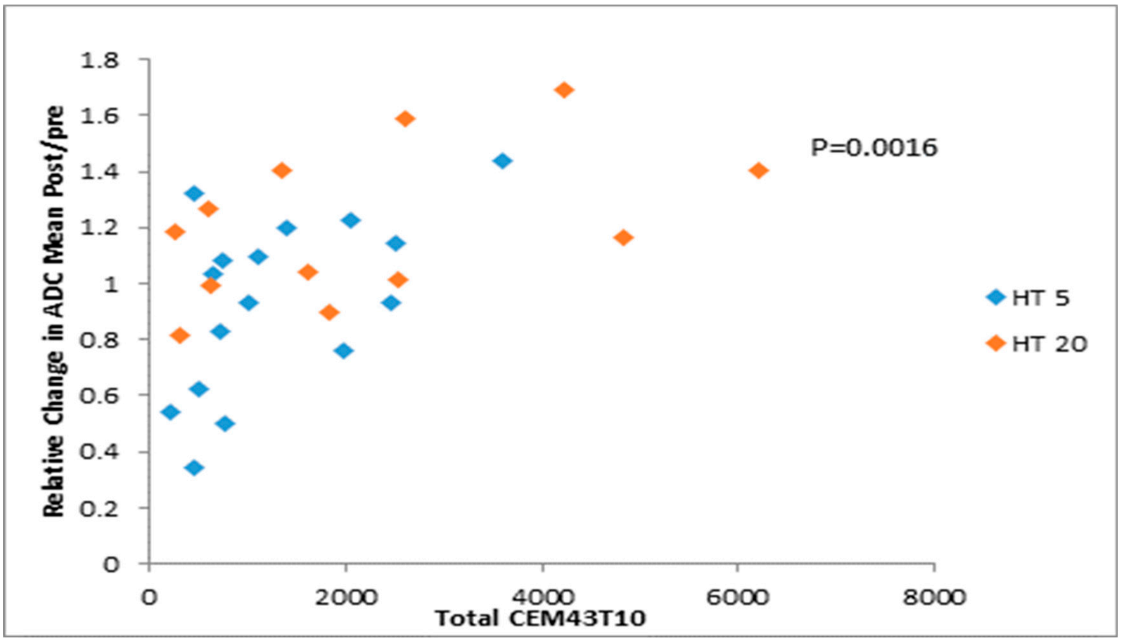

Figure S1. Total CEM43T10 vs. Relative Change in ADC Mean Post/Pre. There is a significant positive correlation between total $CEM43T_{10}$ and relative change in ADC. However, the trends are different for the 5FxHT group vs. the 20Fx HT group. The 5FxHT group tended to show no change or a reduction in ADC at low $CEM43T_{10}$, whereas the 20Fx HT group showed an increase in ADC with increasing $CEM43T_{10}$.

Text S1. Supplemental Methods pertaining to Figure 4

An approach for predicting the combined effects of HT and radiation on cell kill in clinical treatments is presented here, based on experimental results for the effects of HT and radiation on Chinese hamster ovary cells (Loshek et al. [138], Sapareto and Dewey [61]).

Loshek et al. [138] carried out experiments to determine the dependence of cell kill on both the radiation dose (3 Gy) and the number of minutes of exposure to heating at 42 °C. The results were fitted approximately by a function of radiation dose D in Gray and heating time t^* in minutes at 42 °C, representing the "survival surface." In this approximation, the surviving cell fraction S is described by the equation:

$$-\ln(S) = K_R\, D_1 + K_H\, t_1 + K_I\, D_2\, t_2 \quad \text{(S1)}$$

where $D_1 = \max(D - D_q, 0)$, $t_1 = \max(t^* - t_q, 0)$, $D_2 = \max(D - D_{qI}, 0)$ and $t_2 = \max(t^* - t_{qI}, 0)$. The values of the constants are $K_R = 0.48$ Gy^{-1}, $K_H = 0.018$ min^{-1}, $K_I = 0.0034$ Gy^{-1}min^{-1}, $D_q = 0.74$ Gy, $t_q = 25$ min, $D_{qI} = 1.4$ Gy and $t_{qI} = 5$ min.

Sapareto and Dewey [61] examined the dependence of cell kill on temperature in the range 41.5 to 46.5 °C. They expressed the effect of heating for a period t min at a temperature T °C in terms of the period t_{CEM43} that would produce an equivalent effect at 43 °C, where

$$t_{\mathrm{CEM43}}/t = R^{43-T} \quad \text{(S2)}$$

and $R = 0.5$ for $T > 43$, $R = 0.25$ for $T < 43$. This formula represents an exponential increase in the effect of heating with increasing temperature. The change in the coefficient R at 43 °C results in a steeper temperature dependence below 43 °C. It follows that

$$t^* = 4t_{\mathrm{CEM43}} \quad \text{(S3)}$$

where t^* is the equivalent period of heating at 42 °C, and therefore

$$t^* = 4t\ R^{43-T} \quad \text{(S4)}$$

Equations (S1) and (S4) allow prediction of surviving cell fraction as a function of radiation dose and period and temperature of hyperthermia. These equations were applied at each pixel in the images in Figure 4 to predict the corresponding survival fraction under the assumed conditions (heat alone, radiation alone, and combined heat and radiation).

Text S2: Supplemental Materials related to Figure 5

Effect of HT-induced radiosensitization on tumor control probabilty (TCP)

Using the above model parameters, one can model the change in the number of viable clonogens, N, and thus the Poisson tumor control probability (TCP), where TCP is defined:

$$\mathrm{TCP} = e^{-N} \quad \text{(S5)}$$

As a base case we will consider the situation in which a tumor with initial clonogen number, N_0, is treated with radiation therapy 5 days/week, with concurrent hyperthermia applied just prior to the first day of radiation therapy each week of treatment, i.e., during the first week of treatment, radiation is delivered on days 1, 2, 3, 4 and 5 and no radiation is given on days 6 and 7, while hyperthermia is administered only on day 1 only The same sequence is repeated for the remaining weeks of that course of treatment.

We can then use Equation (S1) above with a daily radiation dose, D, of 2 Gy and a t* of 45 min, to serially calculate the daily surviving cell fraction, S. For the purposes of illus tration we will consider two cases—a) an initial clonogen number of 2 × 10^8 cells and a 6-week course of radiation therapy and b) an initial clonogen number of 6 × 10^9 cells and a 7-week course of radiation therapy:

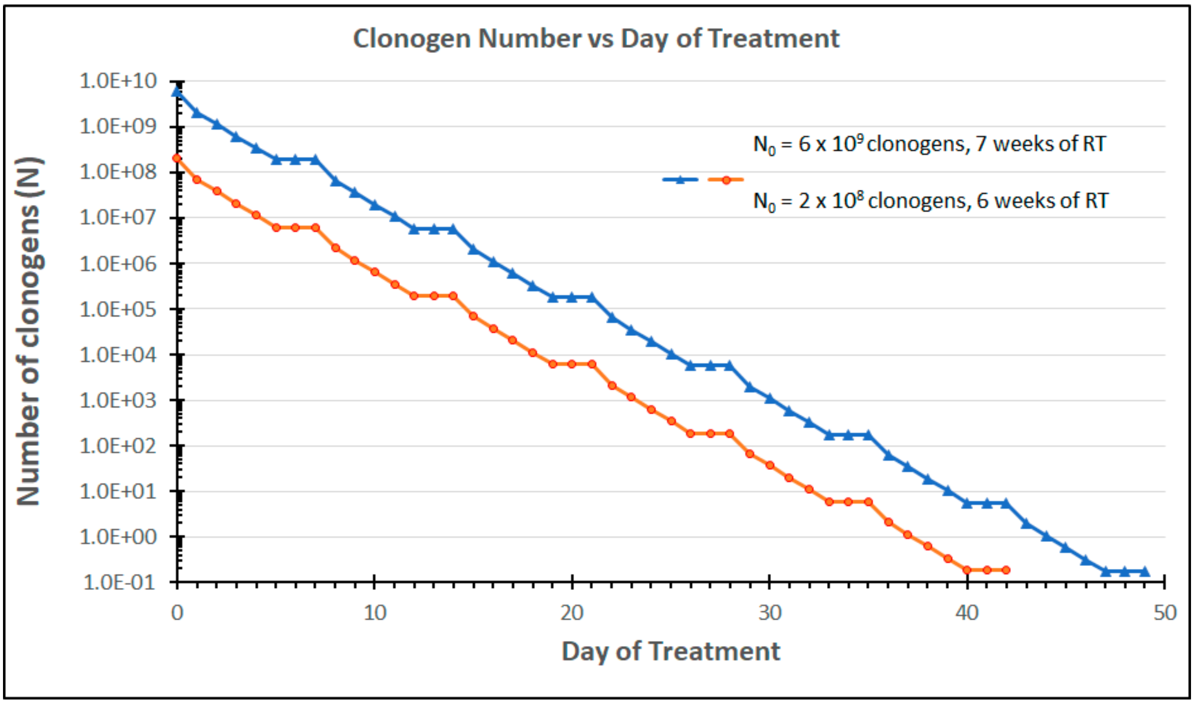

Figure S2. Predicted clonogenic survival vs. day of treament, taking into account heat radiosensitization. The effect of 1 HT per week on surviving fraction is readily observed.

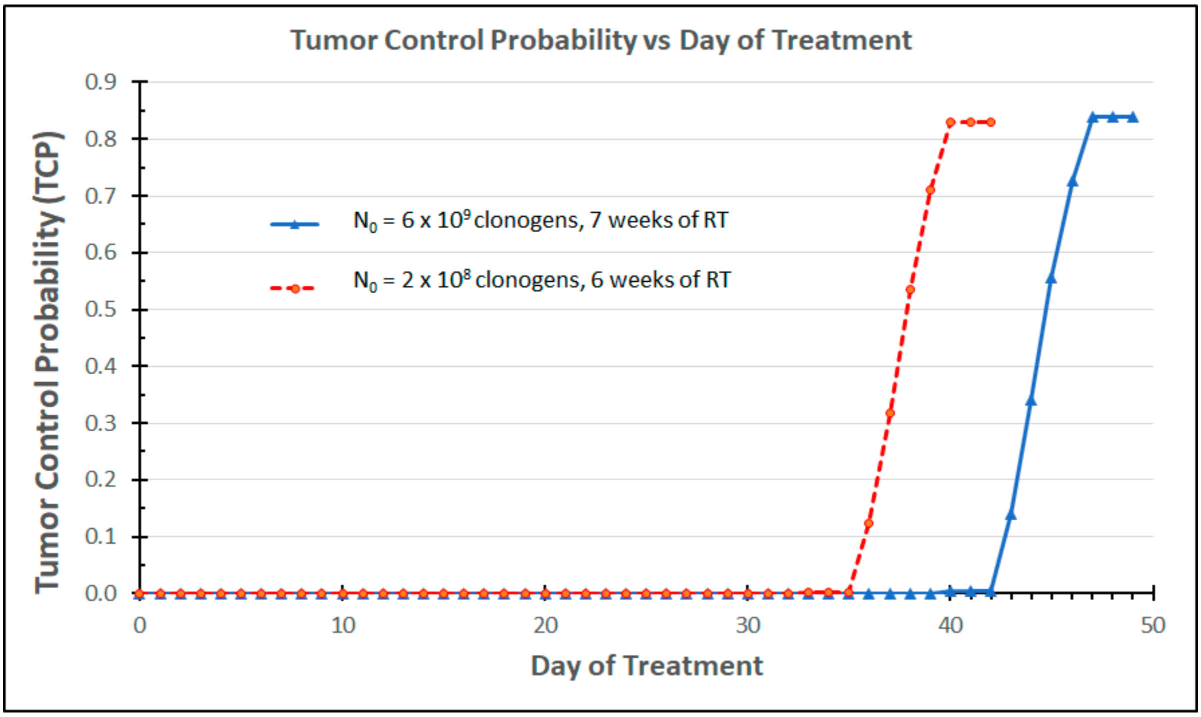

Figure S3. Predicted tumor control probability vs. Day of Treatment. This figure depicts the theoretical tumor control probability vs. day of treatment for the two scenarios shown in Figure S2.

Effect of Heat-Induced Reoxygenation and Hypoxia on TCP

We will now consider the case in which we eliminate the term reflecting heat-induced radiosensitization, K_L, in Equation (S1) and introduce the construct of two compartments into the model—a normoxic compartment, with the surviving fraction following each treatment, S_{Ox}, calculated from:

$$-\ln(S_{Ox}) = K_R D_1 + K_H t_1 \quad \text{(S6)}$$

and a hypoxic compartment with the surviving fraction following each treatment, S_{Hyp}, calculated from:

$$-\ln(S_{Hyp}) = K_R D_1/OER + K_H t_1 \quad \text{(S7)}$$

where OER is the oxygen enhancement ratio.

We will utilize an OER of 2.5 for the remainder of the calculations. Incorporating a permanently hypoxic fraction in a model of this type will severely compromise the ability to control tumor. For example, using Equations (S6) and (S7) with a starting hypoxic fraction of 0.50, $N_0 = 2 \times 10^8$ and a 6-week course of radiation therapy, over 8000 clonogens remain at the end of therapy, yielding a TCP of essentially 0. For the purpose of constructively exploring the effect of reoxygenation, we will arbitrarily increase from 0.48 Gy^{-1} to 1.187 Gy^{-1}, which yields an identical TCP of 0.83 compared to the base case.

We then further modify the model to permit a shift in cells from the hypoxic to the normoxic compartment induced by hyperthermia, on the day following a hyperthemia treatment (day i + 1):

$$N_{Hyp,\,Day\,i} = (1 - f)\, SF_{Hyp} N_{Hyp,\,Day\,i-1}$$
$$N_{Ox,\,Day\,i} = SF_{Ox} N_{Ox,\,Day\,i-1} + f\, SF_{Hyp} N_{Hyp,\,Day\,i-1} \quad \text{(S8)}$$

where f is the fraction of hypoxic cells switching into the normoxic compartment. Note that negative f values reflect a switch from normoxic to hypoxic.

Using the above formulations, TCP from −0.5 to 0.5 were calculated, yielding data shown in Figure 5 of Main text.
